# Acute stress response on Atlantic salmon: a time-course study of the effects on plasma metabolites, mucus cortisol levels, and head kidney transcriptome profile

**DOI:** 10.1007/s10695-022-01163-4

**Published:** 2022-12-27

**Authors:** Angelico Madaro, Jonatan Nilsson, Paul Whatmore, HyeongJin Roh, Søren Grove, Lars H. Stien, Rolf Erik Olsen

**Affiliations:** 1 Institute of Marine Research, NO-5984 Matredal, Norway; 2 Department of eResearch, Queensland University of Technology, GPO Box 2434, Brisbane, QLD 4001 Australia; 3 Fish Health Group, Norwegian Veterinary Institute, 1433 Ås, Norway; 4 Department of Biology, Norwegian University of Science and Technology, 7491 Trondheim, Norway

**Keywords:** *Salmo salar*, Parr, Acute stress, Plasma ACTH, Plasma cortisol, Mucus cortisol, Plasma ions, Head kidney transcriptome profile

## Abstract

Farmed Atlantic salmon (*Salmo salar*) welfare and performance can be strongly influenced by stress episodes caused by handling during farming practices. To better understand the changes occurring after an acute stress response, we exposed a group of Atlantic salmon parr to an acute stressor, which involved netting and transferring fish to several new holding tanks. We describe a time-course response to stress by sampling parr in groups before (T0) and 10, 20, 30, 45, 60, 120, 240, 300, and 330 min post-stress. A subgroup of fish was also subjected to the same stressor for a second time to assess their capacity to respond to the same challenge again within a short timeframe (ReStressed). Fish plasma was assessed for adrenocorticotropic hormone (ACTH), cortisol, and ions levels. Mucus cortisol levels were analyzed and compared with the plasma cortisol levels. At 5 selected time points (T0, 60, 90, 120, 240, and ReStressed), we compared the head kidney transcriptome profile of 10 fish per time point. The considerably delayed increase of ACTH in the plasma (60 min post-stress), and the earlier rise of cortisol levels (10 min post-stress), suggests that cortisol release could be triggered by more rapidly responding factors, such as the sympathetic system. This hypothesis may be supported by a high upregulation of several genes involved in synaptic triggering, observed both during the first and the second stress episodes. Furthermore, while the transcriptome profile showed few changes at 60 min post-stress, expression of genes in several immune-related pathways increased markedly with each successive time point, demonstrating the role of the immune system in fish coping capacity. Although many of the genes discussed in this paper are still poorly characterized, this study provides new insights regarding the mechanisms occurring during the stress response of salmon parr and may form the basis for a useful guideline on timing of sampling protocols.

**Supplementary Information:**

The online version contains supplementary material available at 10.1007/s10695-022-01163-4.

## Introduction

Animals that are exposed to a stressor enter a state of *emergency* (Wingfield et al. 1998; Schreck and Tort 2016). The response to acute stress consists of a set of physiological and behavioral changes that aim to keep the allostatic balance of the animal and maximize survival. In this condition, the body activates a series of adaptive processes (Sterling and Eyer 1988; Sterling 2012). The brain, after the recognition of a real or perceived threat, initiates a response that integrates several factors such as experience, memories, expectation, and re-evaluation of needs in anticipation of physiological requirements (Schulkin and Sterling, 2019; Madaro et al. 2020). A primary response involves triggering of the sympathetic nerve fibers, which innervate the chromaffin cells and subsequently stimulate the release of catecholamines via cholinergic receptors (Winberg et al. 2016). Because catecholamines are stored in chromaffin cells, their release following stress is rapid and the circulating levels of these hormones increase immediately. Following the rapid release of catecholamines, the activation of the hypothalamus-pituitary gland-interrenal gland (HPI) axis induces production and systemic release of cortisol (Wendelaar Bonga, 1997).

The head kidney of teleost fish has a wide spectrum of functions including hematopoietic, immune, and endocrine signaling pathways that are heavily influenced by both the sympathetic and HPI axis once activated by stressors. An acute stressor may enhance dendritic cell, neutrophil, macrophage, and lymphocyte trafficking, maturation, and function which can have an overall stimulating effect on the immune system and the innate and adaptive immune responses (Dhabhar 2008; Aballai et al. 2017; Martorell Ribera et al. 2020). In addition to cortisol and catecholamine, the combination of other stress hormones released from the HPI axis may play a great role in the stress response (Guo and Dixon, 2021). For example, corticotropin-releasing hormone (CRH), adrenocorticotropic hormone (ACTH), and alpha-melanocyte-stimulating hormone (α-MSH) have demonstrated immunoenhancing activities (Watanuki et al. 2003; Castillo et al. 2009; Feng et al. 2022). Conversely, the immune system can be suppressed or dysregulated during periods of prolonged stress, particularly when the allostatic load becomes elevated, and the organism’s available energy becomes not enough to sustain the stress response (Dhabhar and Mcewen 1997; Dhabhar 2008).

Since cortisol must be produced before release from interrenal cells, there is a lag of several minutes before appearing in systemic circulation. As analysis of cortisol is easy and affordable, it has become the most commonly used practical indicator of the stress response in fish (Barton 2002; Kristiansen et al. 2020). The knowledge on how the cortisol production is regulated on a molecular level and how it is affected by environmental factors is however still poorly described. Variation in stress response may occur between different species (Balasch and Tort, 2019). It is well known that the cortisol time-course release varies between rainbow trout (*Oncorhynchus mykiss*), Atlantic salmon *Salmo salar* (Olsen et al. 2002), sea bream *Sparus aurata* (Arends et al., 1999), common carp *Cyprinus carpio* L. (Nematollahi et al. 2009), or Atlantic cod (*Gadus morhua*) (Olsen et al. 2008). For example, in Atlantic cod, the plasma cortisol response following chasing peaks at 60–80 ng/ml remains high for more than 8 h (Olsen et al. 2008), whereas cortisol levels peak at more than 200 ng/ml in Atlantic salmon exposed to the same regime that is subsequently reduced by 75% within 4 h (Olsen et al. 2002). However, the amplitude and duration of cortisol response is dependent on various environmental factors like temperature (Varsamos et al. 2006; Yarahmadi et al. 2016; Madaro et al. 2018), previous exposure to the same stimuli (habituation) (Madaro et al., 2015, 2016), and life history, i.e., salmon parr vs smolt (McCormick et al. 1998; Madaro et al. 2016).

The measurements of cortisol level in the mucus have been suggested as an alternative of blood sampling by being a more minimal/non-invasive method to assess stress, which does not require wounding or killing the fish. While there are no evidences of local cortisol production in fish skin (Gozdowska et al., 2022), skin mucus cortisol levels have been shown to reflect acute stress responses in fish exposed to stressors and to correlate positively to plasma levels (Bertotto et al. 2010; De Mercado et al. 2018; Carbajal et al. 2019). However, in order to study and compare the stress responses in fish, it is required to gather more information about the temporal pattern of post-stress cortisol peak in mucus.

To gain better understanding of what mechanisms are underlying these differences in stress response, it is essential to understand the physiological changes and molecular mechanisms that regulate the response. Here we describe changes and the timing of the stress response of Atlantic salmon parr after being exposed to an acute stressor: netting and transfer to new holding tanks. The fish were sampled sequentially (plasma for ACTH, cortisol, and ions, mucus for cortisol and head kidney for transcriptomics) to obtain a time-course response. A subgroup of fish was also subjected to the same stressor for a second time to assess their capacity to respond to the same challenge again within a short timeframe.

## Materials and methods

### Experimental fish and set-up

Atlantic salmon parr eggs were obtained from a commercial farm (Aqua Gen AS, Trondheim, Norway), hatched at the Institute of Marine Research at Matre (Masfjorden, Norway). Juvenile were kept in an indoor circular tank until parr stage (3-m diameter, 10,000 L). When they had reached an average fork length of 22.51 ± 2.18 cm (mean ± SE) and body weight of 142.29 ± 38.87 g, 120 fish were transferred into two 1 m^2^ tanks, 60 fish in each, filled with 400 L freshwater at 12 °C and let to acclimate for 2 weeks before the trial started. Each tank was covered by a lid furnished with two neon lights programmed to illuminate the tanks under a light regime of 12:12 L:D. The fish were fed ad libitum with Nutra Olympic 2 mm (Skretting, Norway) delivered continuously throughout the 24-h cycle by automatic feeders (Arvo-Tec T drum2000, www.arvotec.fi).

### Stress exposure and sampling

At the start of the trial, 5 fish were netted from each tank unstressed (10 fish total, time 0). The rest of the fish were subjected to a stressor consisting of netting all remaining fish in both tanks while the water levels were lowering and randomized by transferring them into the same transport tank furnished with wheels (0.7 × 0.7 × 0.3 m^3^; crowding density about 11.2 kg/m^3^). The transport tank was then pushed into the next-door room where fish were distributed into eleven new tanks, equal to the former two tanks, until sampling. To minimize the duration of distributing the fish to the experimental tanks, at least ten individuals were rapidly scooped with the net from the transport tank and lifted over to the experimental tank, and the procedure was immediately thereafter repeated for the next tank until all fish were distributed. With a team of personnel scooping fish, distributing the fish into the eleven tanks took less than 45 s. The whole procedure of netting, transport, and distribution into new tanks took 7 min, making the difference in stress duration between groups minimal. In order to study the stress response, 10 fish in each of the respective tanks were sampled at 10, 20, 30, 45, 60, 90, 120, 240, 300, and 330 min post-stress. The fish in one of the tanks were netted at 240 min post-stress and subjected for a second time to the same stressor, thus netted to the transport tank and out again into a new tank of the same type. This group was sampled 60 min after the second stress event (ReStressed).

The fish were sacrificed by an overdose of metacain (500 mg/l, FINQUEL vet., ScanAqua AS, Årnes, Norway) buffered 1:1 with sodium bicarbonate which rendered them completely motionless (no opercular movement) within 10 s of immersion. Each fish was then taken from the anesthetic bath by holding it from the tail for 10 s with forceps in order to allow most of the water to drip off. The fish body was then covered with white precision wipes (KIMTECH Science, Kimberly-Clark® Professional, UK) to absorb the liquid part of the mucus. The soaked wipes were carefully removed by forceps and transferred to Eppendorf tubes (Eppendorf, Germany) which were previously fitted with the top half of a cut 100-μl micropipette tip. The tube was then centrifuged at 13,000 rpm for 5 min to separate the mucus liquid from the wipes which were retained by the micropipette tip. Then the mucus was collected and stored at − 80 °C until further analysis. Mucus samples were not collected for the 10-, 20-, and 45-min time point groups due to the short time available between the sampling points.

Thereafter, fork length and body mass were recorded for each individual fish. Blood was collected using 2-ml heparinized syringes fitted with a 23-G needle and the plasma was separated immediately by centrifugation at 13,000 rpm at 4 °C for 3 min, then frozen on dry ice and stored at − 80 °C until biochemical and cortisol analyses were performed. Head kidneys were collected from the following groups: 0 and 60, 90, 240 and from the ReStressed group. The head kidney samples were frozen immediately on dry ice and stored at − 80 °C until RNA isolation. The sex of each fish was recorded.

### Plasma and mucus analyses

The plasma samples were thawed on ice and the concentrations of plasma ACTH and cortisol were measured by an enzyme-linked immunosorbent assay (ELISA). For each time point, 8 or 9 (for the 10 and 20 min post-stress groups) samples were analyzed for ACTH. The 120- and 330-min groups were not analyzed by ACTH assay. For the ACTH hormone assay, 50 μl of plasma per samples was assessed by a Fish ACTH kit (Cat.no.CSB-E15926Fh, Cusabio Biotech, Houston, TX), while 20-μl subsamples were used for the cortisol assay (standard range: 20 to 800 ng/ml, RE52061 IBL-International, Hamburg, Germany).

The cortisol level in mucus was also quantified by the ELISA method using a Cortisol free in Saliva ELISA kit (Cat.no. DES6611, DemeditecDiagnostics GmbH, Germany). Fifty microliters of mucus was used for analysis, and all procedures followed the manufacturer’s guidelines.

Plasma osmolality was measured by freeze point determination in 20-μl plasma subsamples with a Fiske 210 Micro-Sample Osmometer (Advanced Instruments). The concentrations of other reported plasma parameters, including pH, lactate, glucose, Na^+^, Cl^−^, and Ca^2+^, were measured from 65-μl subsamples with an ABL90 FLEX blood gas analyzer (Radiometer).

### RNA extraction and sequencing

Each head kidney was carefully homogenized before RNA extraction using a Precellys 24 homogenizer and ceramic beads CK28 (Bertin Technologies, Montigny-le-Bretonneux, France). Total RNA was extracted from the head kidney samples using the BioRobot EZ1 and QIAzol Lysis Reagent, with DNase treatment step (Qiagen, Germany). RNA yield was quantified with a NanoDrop® ND-1000 UV–Vis spectrophotometer (NanoDrop Technologies, Wilmington, DE, USA) and RNA integrity assessed with a Bioanalyzer 2100 RNA 6000 Nano Kit (Agilent Technologies, Santa Clara, CA, USA). A 260/280 nm absorbance ratio of 1.8–2.0 indicates a pure RNA sample. All samples had an RNA integrity number (RIN) > 9.4. Library preparation and paired-end RNA-sequencing were carried out at the Norwegian High-Throughput Sequencing Centre (www.sequencing.uio.no). Briefly, libraries were prepared with the TruSeq Stranded mRNA kit from Illumina (San Diego, CA, USA) which involves Poly-A purification to capture coding as well as several non-coding RNAs. The prepared samples were then sequenced on a NovaSeq S1 Flowcell sequencer (Illumina) at an average depth of 50 million reads per sample using a read length of 150 base pairs and an insert size of 420 base pairs.

### Data availability

Sequence data files, in fastq file format, has been uploaded to the Sequence Read Archive (SRA: National Center for Biotechnology Information) under the BioProject number PRJNA788623.

### Statistical analyses

ACTH, cortisol, and ion values are represented as dot plots with mean values and standard error of mean (*N* = 8–9 for the ACTH; *N* = 10 for the other parameters). Changes in plasma and mucus parameters as a function of minutes post-stress were analyzed with cubic, quadratic, or linear regression, preferring the lower order model if the higher order terms did not fit the data significantly better. In case of significant change with minutes post-stress, the model is shown in the plots. Direct comparisons of each post-stress group with the pre-stress (T0) control group for the individual parameters were done by one-way ANOVA and uncorrected Fisher’s LSD test. Differences were considered to be statistically significant at *P* < 0.05. Plasma and mucus statistical analyses were carried out using GraphPad Prism (version 6 for Windows, GraphPad Prism Software, La Jolla, CA, USA).

Paired-end reads were initially trimmed of adapter sequences using cutadapt v 1.18 (Martin 2011) and then quality-trimmed using Trimmomatic v0.39 (Bolger et al. 2014), based on Q30 and a sliding window of 4 bp, with additional 5 bases trimmed from both 5′ and 3′ read ends and removal of reads < 50 bp. Quality-cleaned reads were mapped to the NCBI Atlantic salmon reference genome (ICSASG version2: https://www.ncbi.nlm.nih.gov /assembly/ GCF_000233375.1) using HISAT2 v2.2.1 (Kim et al. 2019). The number of reads that mapped to individual genes (defined by ICSASG v.2 reference genome annotation) was quantified using featurecounts v.2.01 (Liao et al. 2014).

Per-gene differential expression (DE) between stress groups (T0, T60, T90, T240, and ReStressed) was estimated using the R package DESeq2 v1.30.1 (Love et al. 2014). A Wald test identified significantly (*p* < 0.05) DEG, which were then adjusted for false-discovery rates (fdr) using Benjamini-Hochberg (Noble 2009). DESeq2 is specifically designed to accurately identify small expression differences; thus, no fold change restrictions were applied. Identification of GO terms and KEGG (Kyoto Encyclopedia of Genes and Genomes) pathways that were enriched for significantly (fdr-adjusted *p* < 0.05) DE genes was completed using an over-representation test from the R package clusterProfiler (Yu et al. 2012).

The number of shared DE genes between T0 and stress groups were visualized with a Venn diagram, generated by the R package ggVennDiagram (Gao et al. 2021). The number of up- and downregulated genes in each KEGG pathway that had adjusted *p*-value less than 0.05 during the stress was used for calculating Z-score as below formula.

$$\mathrm Z-\mathrm{score}=\frac{\mathrm N\mathrm u\mathrm m\mathrm b\mathrm e\mathrm r\;\mathrm o\mathrm f\;\mathrm u\mathrm p\mathrm r\mathrm e\mathrm g\mathrm u\mathrm l\mathrm a\mathrm t\mathrm e\mathrm d\;\mathrm D\mathrm E\left(\mathrm s\right)-\mathrm N\mathrm u\mathrm m\mathrm b\mathrm e\mathrm r\;\mathrm o\mathrm f\;\mathrm d\mathrm o\mathrm w\mathrm n\;\mathrm r\mathrm e\mathrm g\mathrm u\mathrm l\mathrm a\mathrm t\mathrm e\mathrm d\;\mathrm D\mathrm E(\mathrm s)}{\sqrt{\mathrm N\mathrm u\mathrm m\mathrm b\mathrm e\mathrm r\;\mathrm o\mathrm f\;\mathrm D\mathrm E(\mathrm s)\;\mathrm i\mathrm n\;\mathrm t\mathrm h\mathrm e\;\mathrm p\mathrm a\mathrm t\mathrm h\mathrm w\mathrm a\mathrm y}}$$

All significant KEGG pathways with at least one sampling time point compared to control group (T0) were selected, and the enrichment-network map was applied to visualize Z-score for each pathway and similarity between pathways at all sampling time points using EnrichmentMap App in Cytoscape (ver. 3.7.2). The threshold between pathways based on gene components (similarity) was set to 0.5 (Shannon et al. 2003; Merico et al. 2010).

## Results

### Plasma ACTH levels

**Fig. 1:**
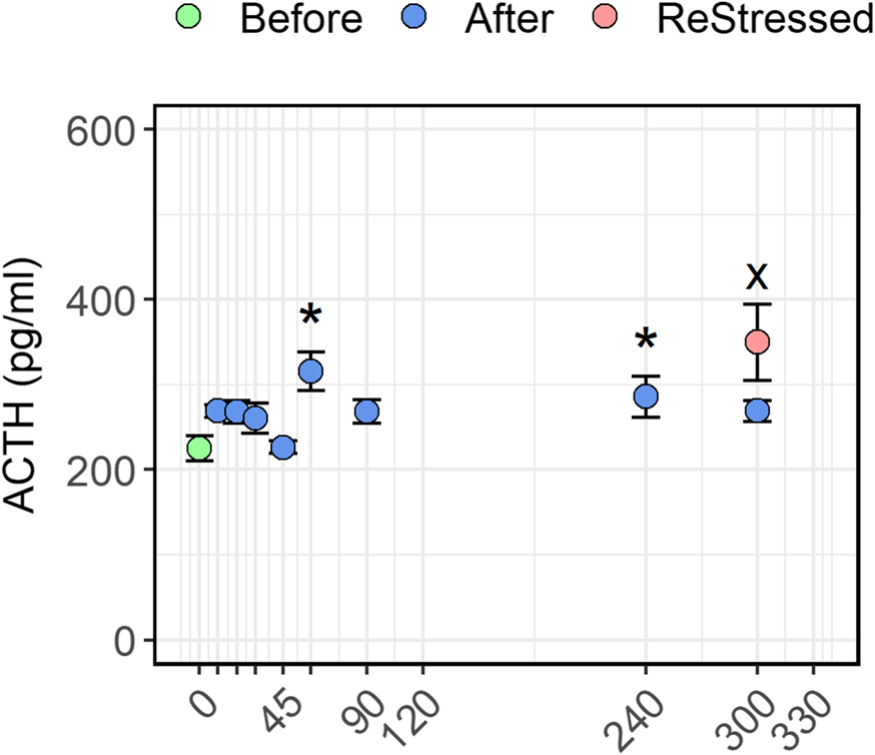
Plasma ACTH levels of Atlantic salmon parr subjected to a stressor consisting of netting transport and transfer into a new tank. Fish were sampled before (0, green dot) and 10, 20, 30, 45, 60, 90, 120, 240, and 300 min post-stress (blue dots). After 240 min post-stress, one group of fish was exposed for a second time to the same stressor and sampled 1 h after (ReStressed, red dot). Values are represented as mean ± SE (*n* = 8–9). Asterisks and the x (for the ReStressed vs time 0 group) show the significance (*P* > 0.05) of each point comparison towards the pre-stress control group (0). There was no significant change with minutes post-stress, and a regression line is therefore not included in the figure

There was no significant change in ACTH levels as a function of time post-stress (*P* > 0.479, Fig. 1). The levels remained stable at around 210 pg/ml, except at 60 min post-stress, when the average level briefly increased to above 310 pg/ml (0 vs 60 min *P* ≤ 0.01) before returning closer to baseline levels at 90 min. At 240 min post-stress, the level of ACTH was marginally higher than in the pre-stress group (*P* = 0.0427). Interestingly, at 300 min post-stress, the ReStressed group has significantly higher ACTH levels (*P* < 0.0001), with some fish responding massively leading to a high standard error of mean.

### Plasma and mucus cortisol levels

**Fig. 2:**
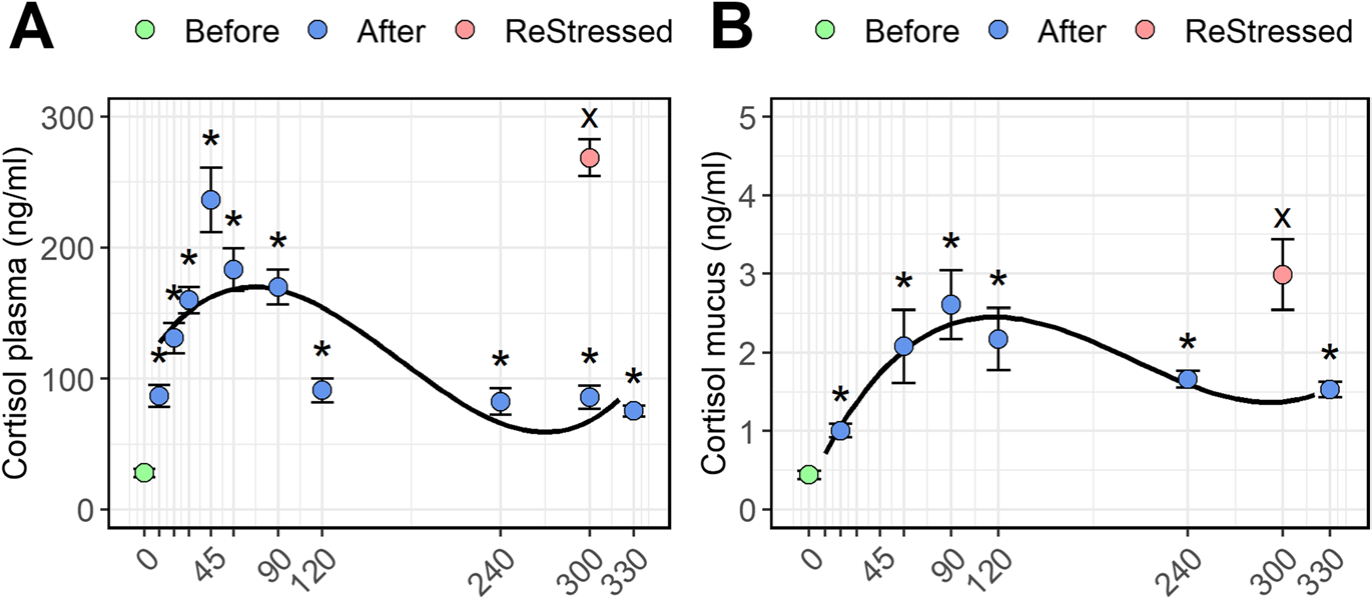
Plasma (**A**) and mucus (**B**) cortisol levels of Atlantic salmon parr subjected to a stressor consisting of netting transport and transfer into a new tank. Fish were sampled before (0, green dot) and 10, 20, 30, 45, 60, 90, 120, 240, and 300 min post-stress (blue dots). After 240 min post-stress, one group of fish was exposed for a second time to the same stressor and sampled 1 h after (ReStressed, red dot). Values are represented as mean ± se (*n* = 10). Asterisks and the x (for the ReStressed vs time 0 group) show the significance (*P* > 0.05) of each point comparison towards the pre-stress control group (0). Significant trends in the post-stress cortisol levels are represented by the regression lines in the figures

The plasma cortisol levels started to rise immediately from 28 ± 3.3 ng/ml (mean ± se) before stress to 87 ± 8.40 ng/ml 10 min post-stress (*P* = 0.001; Fig. 2A) and continued to increase until it peaked at 45 min reaching 236 ± 24.7 ng/ml (*P* < 0.0001). The level then decreased to 183 ± 16.3 ng/ml 60 min post-stress, and even more after 120 min (91 ± 9.25 ng/ml). The cortisol then remained at this level until 330 min post-stress (75 ± 1.5 ng/ml) remaining still significantly higher than the level pre-stress (*P* = 0.0118). Subjecting the fish to a second acute stress led to a higher plasma cortisol level 60 min post-stress than what was found 60 min following the first stress episode (269 ± 14.2 ng/ml; *P* < 0.0001).

Cortisol concentration level in the mucus (Fig. 2B) showed a similar pattern of increase over time (*P* < 0.0001), but the response was delayed compared to plasma levels, peaking at 90 min (2.61 ± 0.4 ng/ml). The level remained relatively high at 120 min (2.171 ± 0.39 ng/ml) and only returned closer to baseline values after 240 min (1.66 ± 0.1 ng/ml). Subjecting fish to a secondary stress triggered a cortisol release that tended to be higher than in the first response at 60 min (2.98 ± 0.45 ng/ml), but the values were not statistically different from the highest peak recorded following the first stress episode.

### Plasma ions and metabolites

**Fig. 3:**
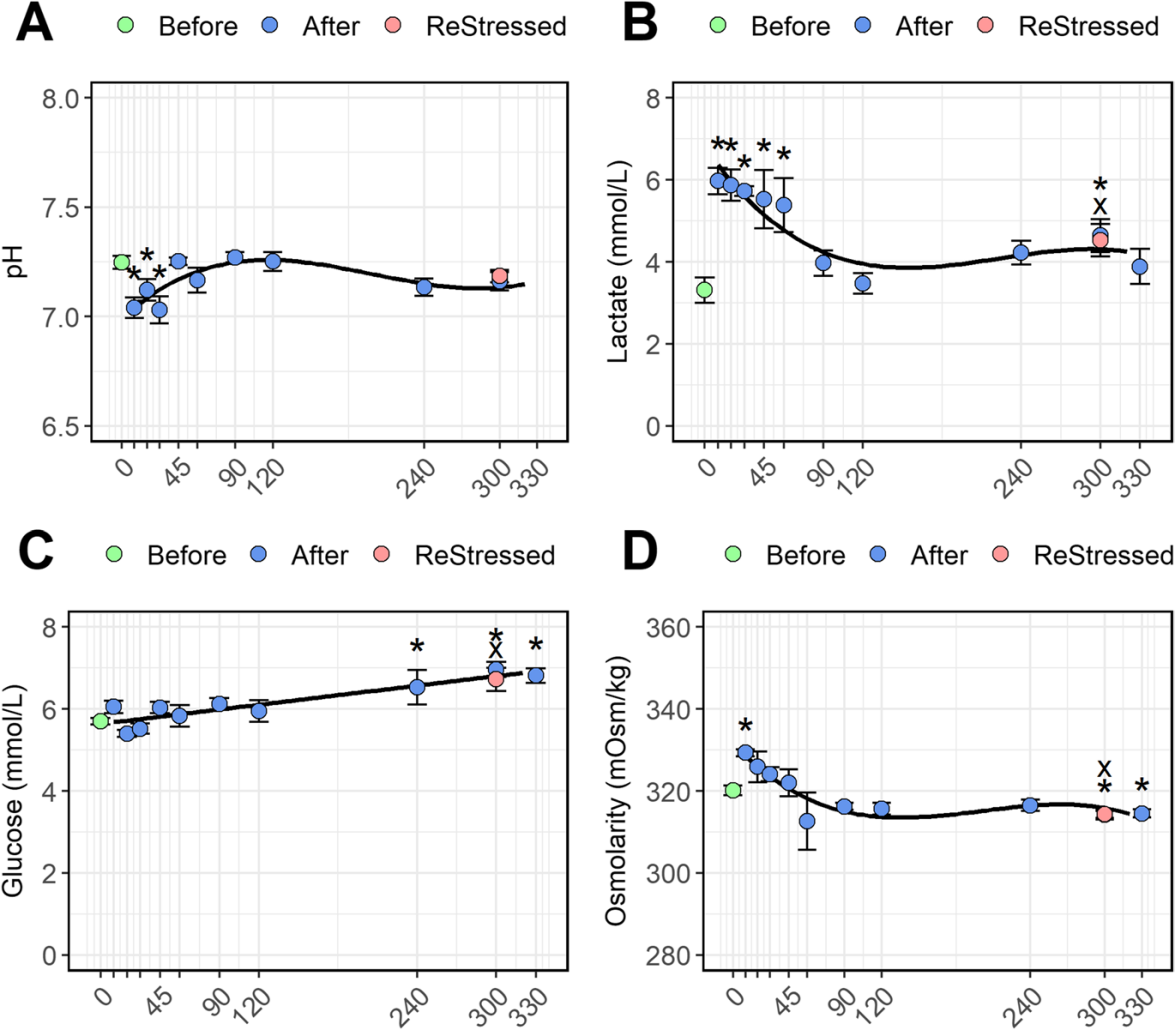
A time variation of plasma pH (**A**), lactate (**B**), glucose (**c**), and osmolarity (**D**) of Atlantic salmon parr subjected to a stressor consisting of netting transport and transfer into a new tank. Fish were sampled before (0, green dot) and 10, 20, 30, 45, 60, 90, 120, 240, and 300 min post-stress (blue dots). After 240 min post-stress, one group of fish was exposed for a second time to the same stressor and sampled 1 h after (ReStressed, red dot). Values are represented as mean ± se (*n* = 10). Asterisks and the x (for the ReStressed group) show the significance (*P* > 0.05) of each point comparison towards the pre-stress control group (0). Significant trends in the post-stress metabolite levels are represented by the regression lines in the figures

**Fig. 4:**
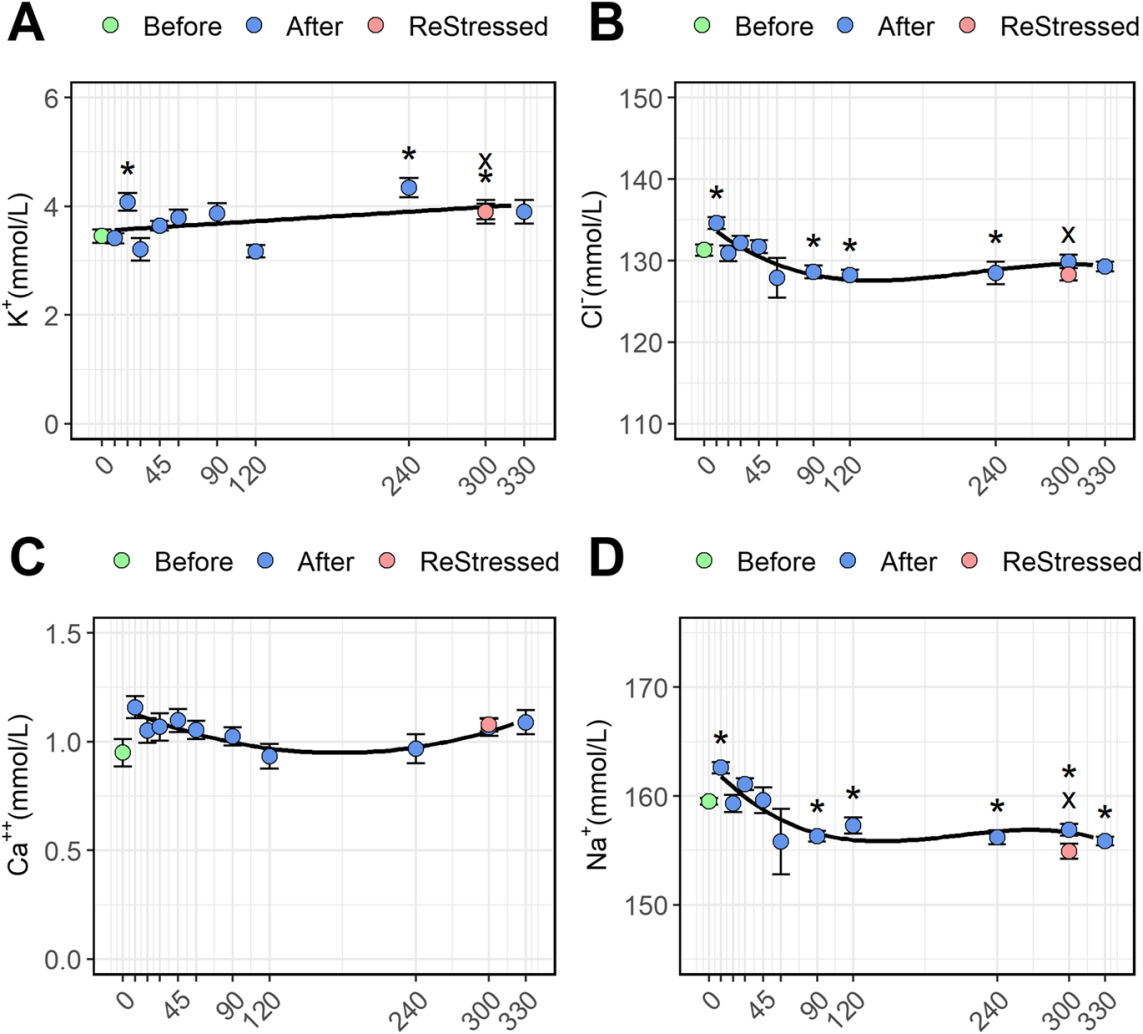
A time variation of plasma K^+^(**A**), Cl^-^ (**B**), Ca^+^^+^ (**C**), Na^+ ^(**D**) ions of Atlantic salmon parr subjected to a stressor consisting of netting transport and transfer into a new tank. Fish were sampled before (0, green dot) and 10, 20, 30, 45, 60, 90, 120, 240, and 300 min post-stress (blue dots). After 240 min post-stress, one group of fish was exposed for a second time to the same stressor and sampled 1 h after (ReStressed, red dot). Values are represented as mean ± se (*n* = 10). Asterisks and the x (for the ReStressed vs time 0 group) show the significance (*P* > 0.05) of each point comparison towards the pre-stress control group (0). Significant trends in the post-stress ions are represented by the regression lines in the figures

The first stress episode caused a significant drop in plasma pH from 7.25 ± 0.02 to 7.04 ± 0.05 (Fig. 3A; *P* = 0.0003) lasting for 30 min post-stress before returning to initial levels. No change was observed in plasma pH 60 min after the second stressor. Lactate levels (Fig. 3B) had increased sharply 10 min post-stress (*P* < 0.0001) and remained high for 45–60 min before returning to basal levels at 90–120 min. Furthermore, lactate was significantly higher than pre-stress levels at 300 min after the first stressor and 60 min after exposure to the second stressor. Plasma glucose (Fig. 3C) grew slowly following the first stress episode, resulting in significantly higher levels than the unstressed group from 240 min post-stress (*P* < 0.0001). The K^+^ concentrations (Fig. 4A) were significantly higher than resting levels only at 20 (*P* = 0.006) min post-stress and from 240 min (*P* = 0.001) to 300 min post-stress (*P* = 0.048) and for the ReStressed group (*P* = 0.048). Like lactate, the plasma osmolarity (Fig. 3D, *P* < 0.0001), Cl^−^ (Fig. 4B; *P* < 0.0001), and Na^+^ (Fig. 4D; *P* < 0.0001) showed a temporary rise in concentration within the first 10 min post-stress: subsequently osmolarity showed a significant drop below pre-stress level from 300 min post-stress (*P* = 0.018), while Na^+^ and Cl^−^ concentration showed a similar drop 90 min post-stress. The stress episode did not affect Ca^++^ levels (Fig. 4C; *P* = 0.112).

### Transcriptome data analyses

A total of 48 Atlantic salmon head kidney samples were sequenced with *n* = 10 per time point (0 min, 90 min, 240 min, re-stress at 300 min), except for 60 min which had *n* = 8 (two tissues were spoiled during processing).

A total of 5832 million paired-end, 150-bp reads were sequenced. On average, 73.35% of reads per sample passed quality filters (≥ Q30, adapters removed and > 50 bp), resulting in 4272 million “clean” reads and an average of 44.72 million reads per sample. These clean reads were mapped to the Atlantic salmon reference genome where an average of 69.03% of reads mapped to gene regions.

### Differential expression and functional enrichment analysis

**Fig. 5:**
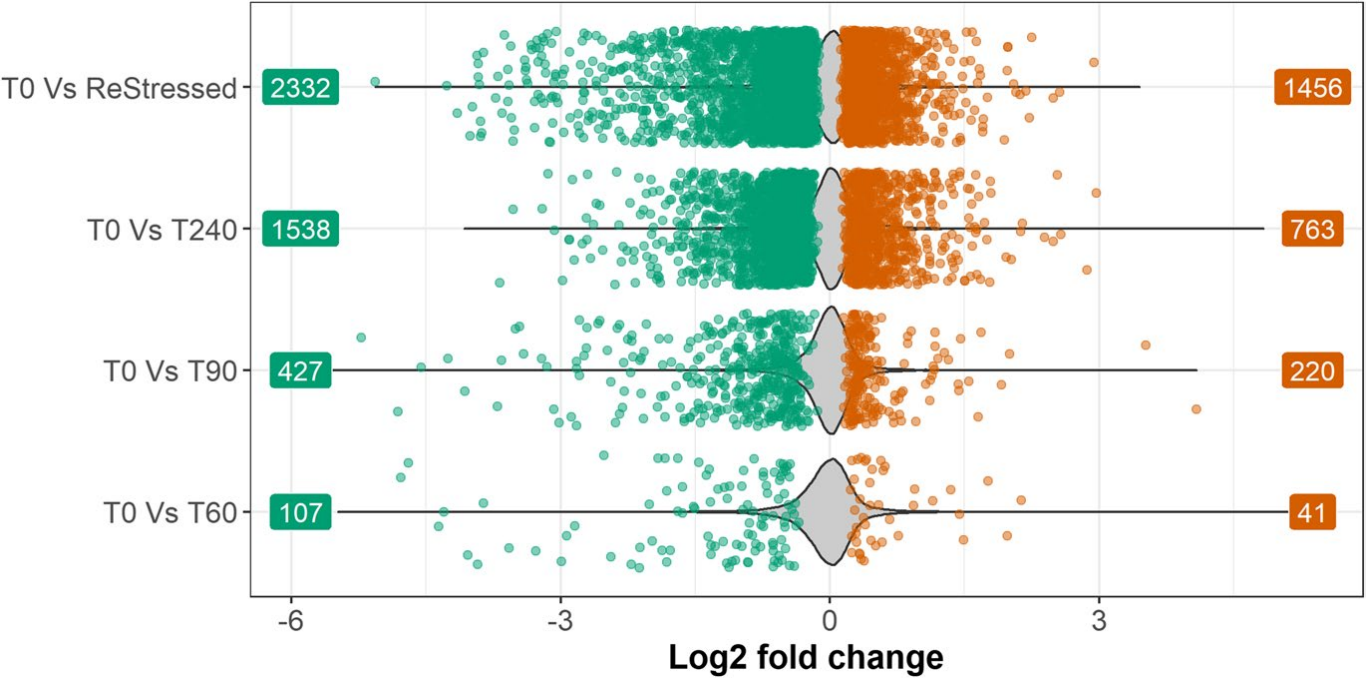
Proportion of upregulated and downregulated genes. Log2 fold change scores of significantly DE genes are represented by scattered dots (green, significantly upregulated; red, significantly downregulated). Violin plot represents non-significant log2 fold change scores

**Fig. 6:**
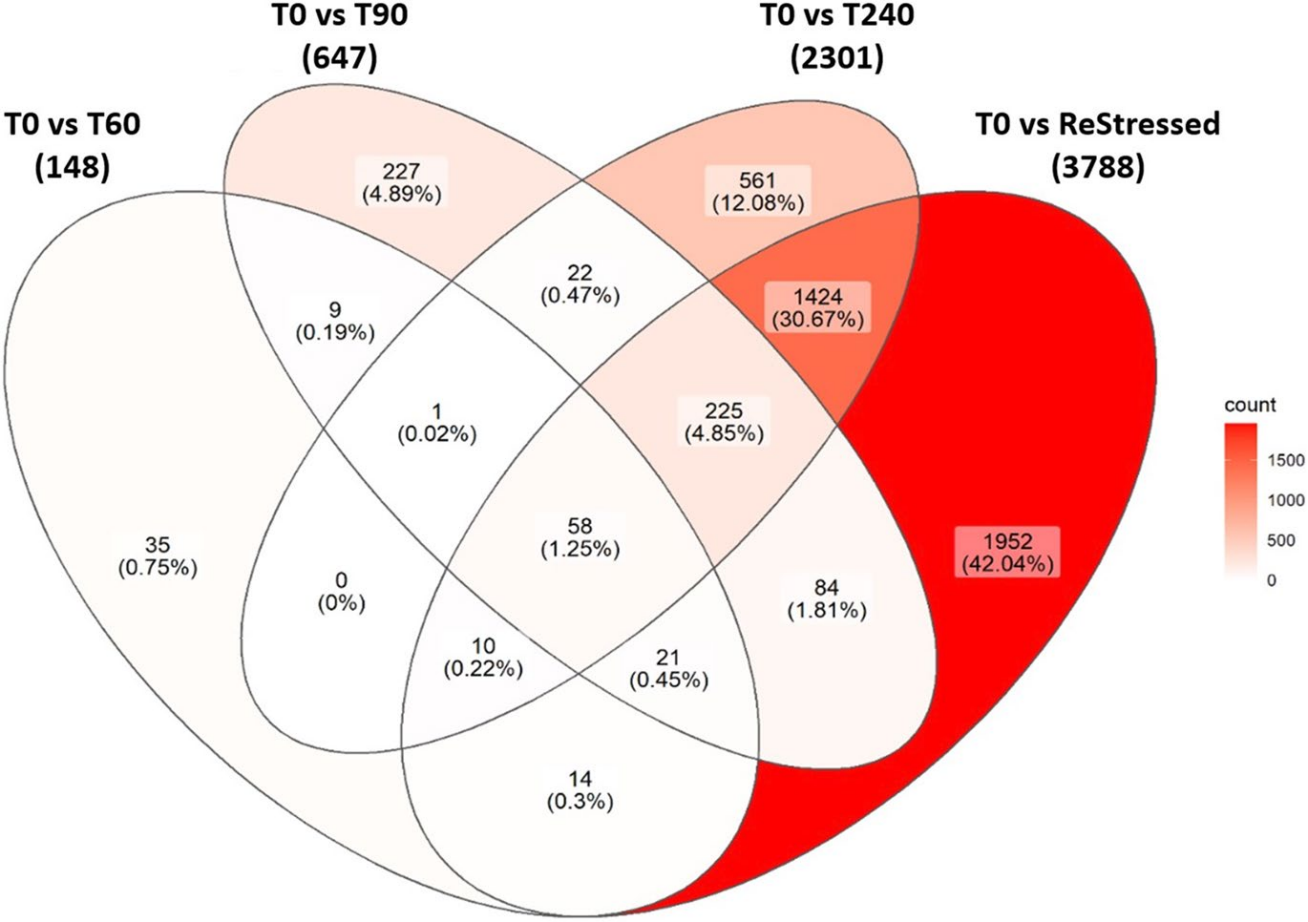
Venn diagram of the different expressed genes (DEGs) in the Atlantic salmon head kidney between pre-stress fish (T0) and 60, 90, 240 min (after stress), and ReStressed groups. The diagram shows the number DEG count and the proportion of the DEG out of all identified DEGs from T0 vs T60, T0 vs T90, T0 vs T240, and T0 vs ReStressed genes occurring in each comparison as well as the DEGs that are shared between several comparisons. The intensity of the background red color in each section indicated the number of DEGs

The entire list of DEGs for each time point is added as file Annex 1. Differential expression comparisons were made as displayed in Fig. 5. Though it was the closest time point to the initial stressor, the number of DEG was low at 60 min post-stress, while they increased successively at 90, 240, and ReStressed (that is 60 min following the second stress episode) minutes following stress. After 60 min (T0 vs T60) from the first acute stressor, only 148 genes were differentially expressed, of which 41 were downregulated and 107 upregulated. At 90 min (T0 vs T90), 427 genes were upregulated and 220 genes downregulated, while at 240 min post-stress (T0 vs T240), the number of DEG was 2301, of which 1538 were upregulated and 763 downregulated. The T0 vs ReStressed fish comparison revealed 2332 upregulated and 1456 downregulated genes (for a total of 3788 DEG). The Venn diagram (Fig. 6) shows the number of genes differentially expressed at each time point following the stress episode, and the changes in DEGs that occurred through the other sampling points. Of the total DEGs, a mere 35 DEGs were expressed only during the first 60 min post-stress, 227 genes were differentially expressed only at 90 min, and 561 at 240 min following the first stress episode. After the second stress episode, 1952 new DEGs were expressed only in the ReStressed group. Interestingly, 14 genes were differently expressed after both T60 and the second stressor (ReStressed). We report these genes in Table 1.

**Table 1:** Genes differentially expressed 60 min after both (T60 and ReStress) stress episodes

T0 vs T60			T0 vs ReStressed				
Log2FC	*p*-value	Adjusted *p*	Log2FC	*p*-value	Adjusted *p*	Description	Entrez. ID
4.78	8.50E-05	0.03	4.27	2.20E-05	0.00083	Neuronal acetylcholine receptor subunit alpha-3	106,592,065
4.3	7.50E-05	0.028	5.07	3.40E-07	2.20E-05	Homeobox protein HMX2-like	106,577,043
3.86	3.00E-05	0.016	4.01	8.60E-06	0.00037	Semaphorin-7A-like	106,605,326
2.52	8.30E-05	0.03	1.91	0.00087	0.015	Mucin-2-like	106,598,491
1.91	0.00012	0.036	1.57	9.10E-05	0.0026	Paired mesoderm homeobox protein 2A-like	106,581,341
0.5	9.80E-05	0.031	0.4	0.00046	0.0094	Papilin a, proteoglycan-like sulfated glycoprotein	106,582,002
− 0.28	8.70E-05	0.03	− 0.21	0.0027	0.034	Zinc finger protein 594-like	106,576,736
− 0.35	7.10E-05	0.028	− 0.23	0.0025	0.033	ADP-ribosylation factor-like 8A	100,195,907
− 0.38	9.70E-05	0.031	− 0.3	0.002	0.028	Parapinopsin-like	106,581,978
− 0.46	0.00015	0.043	− 0.2	0.0013	0.02	Methyltransferase-like protein 6	100,195,690
− 0.47	0.00013	0.039	− 0.38	0.0011	0.018	Major histocompatibility complex class I-related gene protein-like	106,566,091
− 0.6	1.70E-05	0.0099	− 0.39	0.0043	0.048	Calcium-binding mitochondrial carrier protein SCaMC-2-A-like	106,585,451
− 0.93	0.00017	0.047	− 0.74	0.0015	0.022	Zinc finger protein 883-like	106,593,835
− 2.13	4.80E-05	0.022	− 1.46	0.0021	0.028	Hepatocyte cell adhesion molecule-like	106,602,107

### KEGG pathways

Enrichment estimations of Kyoto Encyclopedia of Genes and Genomes (KEGG) are based on an over-representation test, which compares the set of genes within a KEGG pathway or GO-term to the set of genes that are significantly differentially expressed between the comparison groups.

**Fig. 7:**
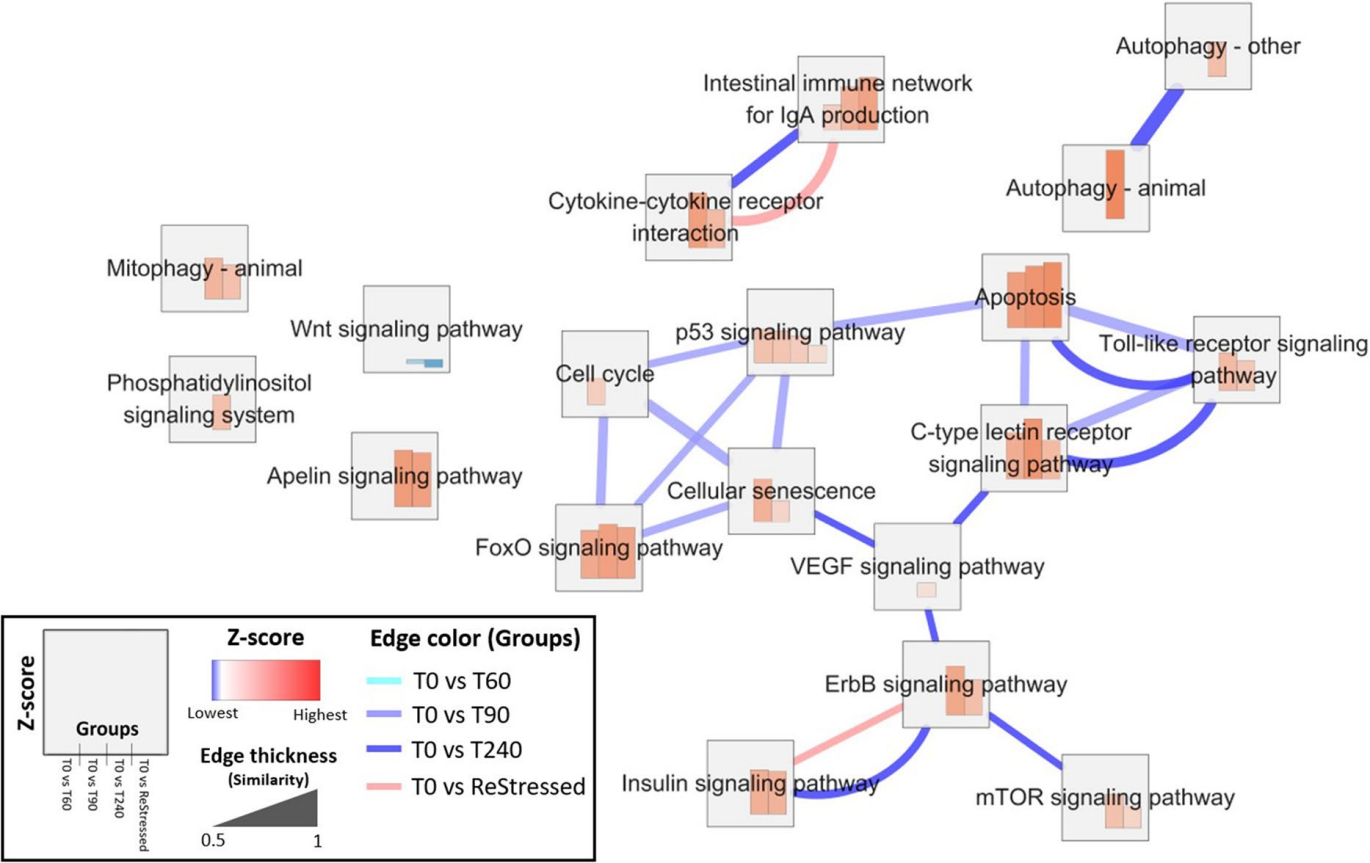
KEEG pathway enrichment analysis belonging. The node (square) divided into four proportions shows Z-scores of T60, T90, T240 and ReStressed group compared to T0. The red bar in each proportion mean show significant pathways with high Z-score (upregulated), and blue bar indicates significant pathway with lower Z-score than 0 (downregulated) at each time point. Edge colors indicate the pathways’ similarity in each groups’ comparison: T0 vs T60, T0 vs T90, T0 vs 240, and T0 vs ReStressed. The edge thickness shows the degree of similarity between pathways; therefore, the thicker lines display the higher number of shared genes between the pathways

There was only one KEGG pathway, the *p53 signaling pathway*, significantly enriched in the comparison between 0- and 60-min groups post-stress (Table 2) with only 5 represented genes. However, the number of enriched KEGG pathways increased considerably for other comparisons. At T90 vs T0, seven additional pathways were enriched, with the most over-represented being the *apoptosis pathway* with 21 genes. Interrenal tissue showed an even greater increase in transcriptome response at 240 min post-stress, with a total of 18 KEGGs enriched pathways. Except for the Cell cycle, all the enriched KEGGs observed at 90 min post-stress were also present at T240 but linked by a much higher number of genes. Finally, following the second stressor, the number of enriched pathways was 13, most of which were the same genes observed at 240 min post-stress, but represented by a higher number of genes. At the last sampling point, the most over-represented pathways were *Wnt signaling pathway* (64 genes), *Cytokine-cytokine receptor interaction* (62 genes), mTOR signaling pathway (59 genes), *FoxO signaling pathway* (58 genes), *Apoptosis* (57 genes), *Apelin signaling pathway* (52 genes), *C-type lectin receptor signaling pathway* (50 genes) *Insulin signaling pathway* (45 genes), *ErbB signaling pathway* (36 genes), *Mitophagy – animal* (33 genes), *p53 signaling pathway* (28 genes), and *Intestinal immune network for IgA production* (16 genes). Figure 7 displays the timeline during the stress response of the KEEG regulation, their functional correlation, and representativity for each time point. In both T60 and ReStressed, not only the number of significant KEGG pathways was small, but few pathways had a high similarity that reflected enough shared genes between pathways. On the other hand, several pathways that showed high similarity among Cell cycle, Cellular senescence, FoxO signaling, p53 signaling, Apoptosis, Toll-like receptor signaling, and/or C-type lectin receptor signaling pathways were observed at T90. Although some pathways such as Apoptosis, Toll-like receptor signaling, and C-type lectin receptor signaling pathway maintained high similarity until T240, more different pathways (e.g., ErbB signaling, Insulin signaling, and mTOR signaling pathway), not highly activated at T90, upregulated and interacted with high similarity at T240.

**Table 2:** Significantly enriched KEGG pathways

	Pathway ID	Pathway description	Adjusted *p*	Gene count
T0 vs T60	sasa04115	p53 signaling pathway	1.7e-02	5
T0 vs T90	sasa04210	Apoptosis	3.0e-05	21
	sasa04218	Cellular senescence	3.1e-02	15
	sasa04068	FoxO signaling pathway	3.1e-02	14
	sasa04620	Toll-like receptor signaling pathway	2.6e-03	13
	sasa04625	C-type lectin receptor signaling pathway	2.1e-02	13
	sasa04110	Cell cycle	3.1e-02	12
	sasa04115	p53 signaling pathway	2.6e-03	11
	sasa04672	Intestinal immune network for IgA production	2.1e-04	9
T0 vs T240	sasa04310	Wnt signaling pathway	7.6e-07	54
	sasa04060	Cytokine-cytokine receptor interaction	1.6e-03	47
	sasa04150	mTOR signaling pathway	1.0e-05	45
	sasa04068	FoxO signaling pathway	1.0e-05	44
	sasa04218	Cellular senescence	1.4e-03	41
	sasa04210	Apoptosis	1.5e-03	37
	sasa04371	Apelin signaling pathway	1.5e-03	37
	sasa04625	C-type lectin receptor signaling pathway	1.2e-04	36
	sasa04140	Autophagy—animal	7.2e-03	35
	sasa04910	Insulin signaling pathway	6.7e-03	33
	sasa04137	Mitophagy—animal	1.6e-04	26
	sasa04620	Toll-like receptor signaling pathway	4.2e-03	26
	sasa04012	ErbB signaling pathway	7.5e-03	24
	sasa04070	Phosphatidylinositol signaling system	1.2e-02	23
	sasa04115	p53 signaling pathway	7.5e-03	20
	sasa04370	VEGF signaling pathway	1.6e-02	18
	sasa04672	Intestinal immune network for IgA production	7.5e-03	12
	sasa04136	Autophagy—other	4.0e-02	9
T0 vs ReStressed	sasa04310	Wnt signaling pathway	5.2e-04	64
	sasa04060	Cytokine-cytokine receptor interaction	2.8e-02	62
	sasa04150	mTOR signaling pathway	1.3e-04	59
	sasa04068	FoxO signaling pathway	1.3e-04	58
	sasa04210	Apoptosis	1.3e-04	57
	sasa04371	Apelin signaling pathway	3.0e-03	52
	sasa04625	C-type lectin receptor signaling pathway	1.3e-04	50
	sasa04910	Insulin signaling pathway	2.8e-02	45
	sasa04012	ErbB signaling pathway	3.3e-03	36
	sasa04137	Mitophagy—animal	1.3e-03	33
	sasa04115	p53 signaling pathway	1.1e-02	28
	sasa04672	Intestinal immune network for IgA production	1.3e-02	16

## Discussion

The primary objective of this study was to closely follow changes and timing of the Atlantic salmon parr physiological response after an acute stressor consisting in crowding and transferring fish into another tank. We used both blood chemistry and head kidney transcriptomics as measurement parameters. Plasma parameters were sampled before and 10, 20, 30, 45, 60, 90, 120, 240, 300, and 330 min post-stress and included measurements of ACTH, cortisol, plasma ions, and metabolites. We also showed, for the first time in Atlantic salmon parr, a timing comparison between plasma and skin mucus cortisol secretion over a period of 330 min post-stress. Changes in head kidney transcriptome profile were assessed at time 0 (before stress), 60, 90, and 240 min after exposure to acute stress. To test the effect of repeated stress, one subgroup was subjected to a second acute stress episode 240 min after the first stress episode (ReStressed). These fish were sampled 1 h later. Transcriptome characterization was performed on approximately 10 fish per time point in order to examine the variability in the stress response that is usually observed between individuals when challenged by a stressor.

While a post-stress sampling point at 60 min for plasma cortisol has been adopted in many stress trials in the temperature range 10–12 ℃ (Madaro et al. 2015, 2016; Delfosse et al. 2021; Djordjevic et al. 2021; Hundal et al. 2021; Ignatz et al. 2021; Lai et al. 2021), the highest values were here found 45 min post-stress, suggesting that sampling at this time point is more likely to catch the peak of the plasma cortisol. Temperature is however an important factor clearly affecting timing and amplitude of cortisol release (Madaro et al. 2018; Samaras et al. 2018; Filipsson et al. 2020; Ignatz et al. 2020) and should be considered in each case. Interestingly, fish sampled 60 min after the first stress episode showed a significant lower level of cortisol compared to those measured 60 min after the second exposure to the same stressor. It can be argued that the higher level of cortisol following the second stressor could be the result of (i) the cumulative sum of the remaining circulating plasma cortisol level of the first stress episode and the cortisol released after the second stress episode, and/or (ii) the results of a sensitization effect due to repeated stress (Belda et al., 2015). For instance, in agreement with the last case, a similar result was observed in a study by Ellis et al. (2004), where the plasma cortisol concentrations in fish subjected to repeated stressors were much higher than in fish stressed only once.

The cortisol levels demonstrated in skin mucus liquid were considerably lower than the levels in plasma, i.e., approximately a hundredth. To date, there are no evidences in literature, nor in the current study, of local cortisol production in fish skin (Gozdowska et al., 2022), while in mammals, evidences reveal that cortisol can be synthesized locally in other organs including primary lymphoid organs, intestine, skin, brain, and possibly heart (Taves et al. 2011; Vukelic et al. 2011; Pondeljak and Lugović-Mihić, 2020). Although a similar mechanism could exist also in fish, the kinetics of the cortisol in mucus resembled to a large degree the kinetics observed in plasma but with a delay of about 45 min. Accordingly with former observation in other fish species (Bertotto et al., 2010; Guardiola et al. 2016), this delay may reflect the rate of cortisol transport to the skin mucus. The data suggest that skin mucus cortisol in fish sampled by the protocol used in this study could be a less invasive alternative to blood samples, especially for fish that are kept alive after sampling. Further studies, including possible effects of temperature and long-term effects of mucus sampling on the fish health, should be done to evaluate mucus cortisol as a possible stress indicator.

Plasma pH dropped just 10 min following the first acute stress and this lasted up to about 45 min post-stress. Rising levels of circulating catecholamines in teleosts trigger, by adrenergic activation, the red blood cell membrane Na^+^/H^+^ exchanger (NHE) (Jensen 2004; Berenbrink 2007). A unique isoform of the red blood cell NHE (termed ß-NHE) is activated owing to ß-adrenoreceptor-mediated mobilization of cyclic adenosine monophosphate (cAMP). The resultant extrusion of H^+^ from the red blood cell will increase intracellular pH ensuring that hemoglobin will deliver O_2_ to peripheral sites in need of the supply (Nikinmaa 1982; Perry and Capaldo 2011). As a consequence, the H^+^ released in the plasma may cause a slight acidification of this last. This mechanism known also as the Bohr effect (Benner et al. 2021) previously described in rainbow trout (*Oncorhynchus mykiss*) (Nikinmaa, 1982) may explain the early plasma pH drop in this study. Also, even if the stress episode was brief, the fish struggle during netting and transfer caused an increase of the plasma lactate during the first 45 min, which also may have affected the plasma pH. Fish under stress or exercise naturally increase the gill ventilation and the functional surface area in order to get higher gas exchange between the gills and the surrounding water. Under these conditions, the heightened blood perfusion in the gill in a fresh (hypotonic) water environment also increases ion loss, e.g., Na^+^ and Cl^−^, across the gills, a phenomenon known as osmoregulatory compromise (Gonzalez and McDonald 1992; Nilsson and Sundin 1998). Interestingly, this effect is observed mostly for the fish sampled 60 min after the first stress episode. The plasma of fish sampled immediately after the first stress episode showed a clear increase of osmolarity and ions (particularly Na^+^ and Cl^−^). Increase in branchial ion loss post-stress is not always reflected by a reduction of plasma osmolarity (Wendelaar Bonga, 1997). Therefore, after an acute stress episode in freshwater fish, blood plasma volume may be decreased as a result of plasma water moving out the circulation and into the tissues. This may lead, as observed in the current study, to a transient increase of plasma osmolarity and electrolytes as previously showed by Okimoto et al. (1994). This phenomenon may also be responsible for the increased plasma lactated concentration measured in the first minutes post-stress. The stress increased the circulating levels of glucose, which become particularly evident only after 4 h post-stress and in the ReStressed group. This delay may be due to the brief nature of the stressor since in other studies with salmonid, where more severe stressors were applied, plasma glucose increased within 1 h after stress (Barton 2000; Pankhurst et al. 2008; Chalmers et al. 2018; Cadonic et al. 2020).

As for most vertebrates, the stress response in fish is modulated by two hormonal systems: the fast system which is controlled by the brain-sympathetic nerves-chromaffin cell axis which triggers release of catecholamines such as adrenaline and noradrenaline. The slower system is controlled by the HPI axis, where ACTH produced by the pituitary gland is released into circulation (Wendelaar Bonga, 1997). The ACTH peptide is considered the major factor responsible for inducing cortisol synthesis and release from interrenal cells in the head kidney (Rodrigues and Sumpter 1983; Raffin-Sanson et al. 2003; Flik et al. 2006). However, our data shows that the circulating levels of plasma ACTH did not increase immediately following stress but rather after 60 and 240 min which is much later than the actual cortisol release that was observed already after 10 min post-stress. Thus, it may appear odd to consider ACTH as the main regulator of the early cortisol production in Atlantic salmon parr subjected to acute stress. A possible candidate to trigger cortisol release may be catecholamines. This hypothesis is discussed below.

Upon reaching the head kidney, ACTH must activate melanocortin receptors 2 (Mc2R) to initiate the synthesis and secretion of adrenal glucocorticoids (Pantel and Cone, 2013). However, to be functional, Mc2r requires an adrenal-specific factor named Mc2r accessory protein (Mrap) (Metherell et al., 2005). In the present study, we found the upregulation *mrap2a* at 240 min (1.59 Log2fc) as well as 1 h following the second stress in the ReStressed group (1.98 Log2fc), possibly with the aim of supporting ACTH-dependent stress-coping mechanisms later following the stress response. In zebrafish (zf), MRAP2 has two paralog genes, i.e., zfMRAP2a and zfMRAP2b (Agulleiro et al., 2010). In the presence of zfMRAP2a, ACTH will attain increased affinity for zfMC4R which is usually only activated by α-MSH (Josep Agulleiro et al., 2013). Therefore, the binding to *mrap2a* may confer to ACTH additional functions including the capacity to target MC4R to modulate the energy balance. This mechanism has previously been suggested in mammals and would be independent from glucocorticoid pathways (Soletto et al., 2019).

In a former study, Rotllant et al. (2006) showed that catecholamines released from chromaffin cells in vitro may have a paracrine effect that stimulates cortisol release. Intra-adrenal interactions are well studied for mammals (Ehrhart-Bornstein et al., 1998) and the stimulatory effect of catecholamines on steroidogenesis has been demonstrated (Haidan et al., 2000). In fish, this mechanism may be species specific, since the stimulatory effect of catecholamines on cortisol release and steroidogenesis has been observed in sea bass (*Dicentrarchus labrax* L.) but not in sea bream (*Sparus aurata*) (Rotllant et al., 2006). Although a possible relationship between catecholamines and cortisol has not been demonstrated in Atlantic salmon, it is a distinct possibility. This means that plasma ACTH may rather contribute to sustain cortisol production later after the stress episode start.

The secretagogue action of catecholamines is mediated by a β-adrenoceptors, which stimulate the release of intracellular cAMP (Rotllant et al., 2006). Interestingly, the parr transcriptome data indicate that the *beta-2 adrenergic receptor-like* was upregulated following stress (1.09 Log2fc) for at least 240 min (0.91 Log2fc), and also after the second stress episode (1.16 Log2fc). Similarly, at 90 min post-stress, several members of the *activation transcription factor* (*atf*)/*cyclic AMP-responsive element-binding protein* (CREB) protein family of transcription factors were activated (Annex 1). For instance, upregulation of *aft-3* expression and cyclic AMP-dependent transcription factor ATF-3-like at 90 (1 and 1.52 Log2fc respectively), at 240 min after stress (0.98 and 1.66 Log2fc respectively), and for the ReStressed group (1.07 and 1.81 Log2fc respectively) is in line with results from an in vitro study on neuronal rat cell culture (Chen et al. 1996). These genes are suggested to perform essential roles in the nervous system including neuronal survival, growth, nerve cell protection, and neurodegeneration (Pai et al., 2018).

A small number of genes, i.e., the 14 genes listed in Table 1, were DE both in the T60 and ReStressed groups. Although the function is not known for all of them, it is worthy to mention the *neuronal acetylcholine receptor subunit alpha-3* (nAChRα3) gene, a protein transmitter–gated cation channel that detects excitatory chemical signals, e.g., acetylcholine (ACh) at the synapses in the nervous system (Karlin, 2002). In fish, the chromaffin cells are innervated by pre-ganglionic sympathetic nerve fibers. The release of acetylcholine ACh by these nerve fibers is thought to be the predominant mechanism underlying catecholamine secretion during acute stress (Perry and Capaldo 2011). The strong upregulation of this gene after every stress episode that resulted up to 4.27 LogFC in ReStressed fish and up to 4.78 Log2FC following the first stress episode makes it a possible marker gene of stress in fish.

In addition, between the most upregulated DEGs, *secretogranin II b* (*sg II*; 2.93 Log2FC) is part of a class of proteins of the granin family which in mammals is co-released during catecholamine secretion from chromaffin cells. As in other granins, SgII 2 may be involved in the packaging of hormones and the formation of secretory granules, and as precursors of several peptides that could be released to exert hormonal effects in autocrine, paracrine, and endocrine manners (Montero-Hadjadje et al. 2007).

The expression of *neuromodulin* and *galanin receptor type 1-like* (*GalR1*) genes were also strongly upregulated at 90 and 240 min after the first stress episode, and for the fish of the ReStressed group. The *neuromodulin* protein in rats has been shown to regulate noradrenalin secretion from chromaffin cells (Grant et al. 1992). GalR1 is a G-protein coupled receptor that has been suggested to modulate the HPA axis during handling of the stress response (Picciotto et al. 2010; Ullrich and Mac Gillavry 2021). Also, several genes involved in the synaptic firing, vesicular traffic, exocytosis, and secretion like were upregulated. Just to mention some of them: synaptotagmin (Syt) Syt1, Syt4, Syt6, and Syt11 which, anchored in vesicle membranes, mediate fast Ca^2+^-dependent exocytosis of synaptic vesicles (Park and Ryu 2018), or Syntaxin-1B (1.97 Log FC) and Synaptophysin (2.98 Log FC) that are essential for synaptic vesicle fusion and neurotransmitter release (Chang et al. 2021; Melland et al. 2021). All together, these observations may suggest that the stress episodes caused activation of the head kidney by the sympathetic connections, while the upregulation of the transcription of those genes may be a requirement to refurbish the proteins employed in the stress response in order to make the fish ready to meet the environmental demands.At 60 min post-stress, we observed a strong activation of several regulatory genes associated with the cell proliferation and differentiation including *cef10* (4.04 Log2FC), *connective tissue growth factor like* (4.7 Log2FC), *thrombospondin 1-like and immediate early response 2* (2.21 Log2FC), and *mucins* (*mucins 2 like*; 2.52 Log2FC). These genes belong to a class of immediate-early genes that in mammals can be inducted by growth factors or oncogenes (Bradham et al., 1991). These immediate early genes are necessary for the G0-G1, transition of the cell cycle (for review, see Rollins and Stiles 1989), and for the activation of mitogen activity post-stress in the head kidney. The strong upregulation of *c-fos* (3.9 Log2FC) and *jun B* (1.09 Log2FC) proto-oncogenes, that are essential for cell cycle regulation, seem to support this hypothesis (Bradham et al. 1991).

The rise in the transcriptional activity 90 min post-stress increased the number the DEGs interconnected to two main types of pathways. On one side, stress changed the expression of pathways connected to apoptosis, p53 signaling, and cellular senescence. On the other hand, enriched KEGGs pathways, like Toll-like receptor signaling, Cytokine-cytokine receptor interaction, FoxO signaling, C-type lectin receptor signaling, and Intestinal immune network for IgA production, suggest the triggering of pathways critical for the regulation of innate and adaptive immunity. These pathways were represented with even more DEGs later at 240 min following the first stress response, which coincided with decreasing plasma cortisol levels.

Yada and Tort (2016) extensively reviewed the effect of acute stress on fish immunity. Acute stress activates the immune response by boosting the fish innate responses, cell production, and mobilization of the Th1 response. Acute stress will therefore stimulate the production of new blood cells in the head kidney, including both erythrocytes and leukocytes, resulting in elevated numbers of circulating leukocytes to be distributed to target tissues. We found upregulation of several genes encoding proteins mainly expressed on the surface of leukocytes that are playing a prominent role in cell migration and antibacterial immunity. These included two genes of the Epidermal growth factor-seven span transmembrane family, the *CD97 antigen-like* gene (1.48LogFC) and *multiple epidermal growth factor-like domains protein* 6 (1.43LogFC) (Leemans et al. 2004; Safaee et al. 2014; Hu et al. 2018). In addition, there was a strong upregulation of the *nuclear factor of activated T cells 1* (1.78LogFC) and *transcription factor AP-1-like* (1.53LogFC) whose proteins attach to DNA and trigger transcription of numerous genes involved in the immune response (Macián et al. 2001). Furthermore, at 240 min, we observed that stress induced a proinflammatory response through upregulation of inflammatory cytokines (see supplementary material). This includes C-type lectin receptor signaling pathway, Cytokine-cytokine receptor interaction, Toll-like receptor signaling pathway genes and above all the Interleukin-17 receptor A (1,13 LogFC) whose protein receptor activation may explain the strong upregulation of the cytokine interleukin-1beta-like gene (IL-1β) (Zenobia and Hajishengallis 2015). We also found an upregulation of the interleukin-7 receptor subunit alpha-like gene important for the proliferation and differentiation of B lymphocyte (Corcoran et al. 1996). Semaphorin-7A-like, one of the genes modulated in both stress events (Table 1), was strongly upregulated in T60 (3.86 LogFC) and ReStressed groups (4.01 LogFC). Studies on mammals have demonstrated the involvement of this gene in both the innate and adaptive immune systems (Xie and Wang 2017).

Fish subjected twice to the same acute stress challenge, i.e., again 4 h after the first stress episode, showed a second peak for cortisol and ACTH release in the plasma as well as an increase in trascriptome activity. We cannot say for sure for ReStressed fish which gene expression are triggered by the first stress episode and which from the second. However, given that the highest cortisol level was observed in the ReStressed group, it is highly possible that 1952 DEGs that are only observed at the ReStressed group may link with an adaptive response following the second stress episode. The KEEG pathways shared between the T0 vs ReStressed groups were less than the ones observed at 240 min, with Autophagy, Toll-like receptor signaling pathway, Cellular senescence, Phosphatidylinositol signaling system, and VEGF signaling pathways undetected after 240 min. On the other hand, all the other KEEGs were still observed at Restressed and all of them represented with a higher number of DEGs.

For the stress response to be adaptive, it must be activated whenever necessary and then switched off when no longer required. The stress-induced transcription in the head kidney can be short-lived and may require a negative feedback to dampen the negative consequences. The cortisol release in this experiment peaked at 45 min post-stress and then started to decrease. The transcriptome data suggests a strong responsiveness with the rising cortisol of several *dexamethasone-induced Ras-related protein 1-like genes* (*Rasd1*, or also known as *Dexras1*). *Rasd1* is a member of the Ras family of monomeric G proteins that was first identified as a dexamethasone-inducible gene in the pituitary corticotrophs cell line AtT20 (Kemppainen and Behrend 1998). Previous studies showed that the peripheral administration of glucocorticoids in rats and mice strongly and rapidly induces Rasd1 expression in several tissue types, including the brain (Kemppainen and Behrend 1998; Brogan et al. 2001; Kemppainen et al. 2003). In the cytoplasm, RADS1 activates Gαi which inhibits the cAMP-dependent pathway by inhibiting adenylyl cyclase. This results in the inhibition of the cAMP-PKA-CREB signaling pathway (Greenwood et al. 2016). In mammalian cells, Rasd1 synthesis appears to be induced by glucocorticoid receptors (GR) due to increased circulating levels of corticosteroids (Greenwood et al. 2016). In the brain, it is synthetized in corticotroph cells and may play a role in the negative feedback loop controlling ACTH secretion (Brogan et al. 2001). It is possible that rasd1 may have similar roles in Atlantic salmon. We found two *rasd1* paralogs (LOC106606458 and LOC106606457) induced by the two stress episodes, upregulated at 60 and 90 min after the first stress, and 60 min after the second stress, coinciding with the highest cortisol levels. However, these paralogues returned to pre-stress condition at 240 min post-stress. On the other hand, a new *rasd1* paralog (LOC106579016) was strongly upregulated from 240 min post-stress and following the second stress episode, while during the same time, two other new paralogues were downregulated (LOC 106,601,919 and LOC 106,601,905). Interestingly the first two *Rasd1* paralogue genes activated after stress, i.e., LOC106606458 and LOC106606457, sit on the same ssa6 chromosome, while the rads1 paralogue that is upregulated later from 240 min post-stress (LOC106579016) is on the ssa 19 chromosome. The last two *Rasd1* paralogues that were downregulated are both situated on the ssa3 chromosome. It is reasonable to think that these paralogues may regulate different pathways and be activated differently in different cell populations. This is the first time that *rasd1* expression is described in Atlantic salmon, and these results showed that *rasd1* could represent a very good candidate as a marker to study negative feedback in Atlantic salmon head kidney, and that further studies are required to better understand its regulating mechanism in fish.

## Conclusions

This time-course study aimed to highlight the physiological changes and molecular mechanisms that occur on plasma metabolites, mucus cortisol levels, and head kidney transcriptome profile of Atlantic salmon parr after being exposed to an acute stressor, consisting of netting and transfer to new holding tanks. One of the most interesting observations is the late rising of ACTH in the blood plasma compared the earlier release of cortisol. This last observation may suggest that early cortisol release could be triggered by a faster mechanism, for instance the sympathetic system. In support of this hypothesis, a very high upregulation of several genes involved in the synaptic firing was both demonstrated in the first and in the second stress episodes. Furthermore, the head kidney transcriptome profile showed that a very few genes were upregulated within the first 60 min post-stress. On the other hand, thereafter, the head kidney transcriptome profile displayed that the acute stress episode induced the transcriptome of several immune-related pathways in support of the stress response. Although many of the genes discussed in this paper require further investigation, we have tried to highlight genes modulated by stress, and/or for example involved in the negative feedback to end the stress response. Finally, the study gives new insight into the mechanisms of stress response in Atlantic salmon and could form the basis for a useful guideline for sampling timeline protocol.

## Supplementary Information

Below is the link to the electronic supplementary material.

Supplementary file1 (XLSX 376 KB)

## Data Availability

The datasets generated during and/or analyzed during the current study are available from the corresponding author on reasonable request.
